# Profiling of Protein Degraders in Cultures of Human Gut Microbiota

**DOI:** 10.3389/fmicb.2019.02614

**Published:** 2019-11-15

**Authors:** Alberto Amaretti, Caterina Gozzoli, Marta Simone, Stefano Raimondi, Lucia Righini, Vicente Pérez-Brocal, Rodrigo García-López, Andrés Moya, Maddalena Rossi

**Affiliations:** ^1^Department of Life Sciences, University of Modena and Reggio Emilia, Modena, Italy; ^2^BIOGEST-SITEIA, University of Modena and Reggio Emilia, Modena, Italy; ^3^Area of Genomics and Health, Foundation for the Promotion of Sanitary and Biomedical Research of Valencian Community (FISABIO-Public Health), Valencia, Spain; ^4^CIBER in Epidemiology and Public Health (CIBERESP), Madrid, Spain; ^5^Institute for Integrative Systems Biology (I^2^SysBio), University of Valencia, Spanish National Research Council (CSIC-UVEG), Valencia, Spain

**Keywords:** gut microbiota, proteolysis, metagenomics, enrichment culture, Enterobacteriaceae

## Abstract

Unabsorbed proteins reach the colon and are fermented by the microbiota, yielding a variety of harmful metabolites. In the present study, a 16S rRNA gene survey identified the bacterial taxa flourishing in 11 batch fermentations with proteins and peptones as the sole fermentable substrates, inoculated with the feces of six healthy adults. Organic acids, ammonia, and indole resulting from protein breakdown and fermentation accumulated in all of the cultures. Analysis of differential abundances among time-points identified Enterobacteriaceae, Burkholderiaceae, and Desulfovibrionaceae (including *Esherichia-Shigella*, *Sutterella*, *Parasutterella*, and *Bilophila*) among the bacteria that especially in the cultures with low inoculation load. Lachnospiraceae and Ruminococcaceae also encompassed many taxa that significantly expanded, mainly in cultures inoculated with high inoculation load, and showed the strongest correlation with the production of ammonium, indole, and *p*-cresol. *Anaerotruncus*, *Dorea*, *Oscillibacter*, *Eubacterium oxidoreducens*, *Lachnoclostridium*, *Paeniclostridium*, and *Rombutsia* were among them. Other Firmicutes (e.g., *Roseburia*, *Ruminococcus*, *Lachnospira, Dialister*, Erysipelotrichaceae, and Streptococcaceae) and many Bacteroidetes (e.g., Barnesiellaceae, Prevotellaceae, and Rickenelliaceae) decreased. Sequences attributed to *Bacteroides*, unresolved at the level of species, presented opposite contributions, resulting in no significant changes in the genus. This study sheds light on the multitude of bacterial taxa putatively participating in protein catabolism in the colon. Protein fermentation was confirmed as unfavorable to health, due to both the production of toxic metabolites and the blooming of opportunistic pathogens and pro-inflammatory bacteria.

## Introduction

The human colonic microbiota is a dense and complex community of commensal microbes, mostly bacteria, gaining energy and nourishment in strict anaerobiosis from non-digestible dietary substrates and host-derived secretions (Scott et al., 2013). Non-digestible oligo- and polysaccharides are the substrate of intestinal saccharolytic bacteria. Carbohydrate fermentation mainly yields short-chain fatty acids (SCFA), which fuel the enterocytes and are major contributors to the maintenance of gut function and to immune homeostasis (Sivaprakasam et al., 2016). Therefore, intestinal saccharolytic catabolism is regarded as beneficial for the maintenance of health and has attracted considerable attention in the last few decades, leading to the development of prebiotic supplements (Gänzle and Follador, 2012; Wilson and Whelan, 2017).

Proteins are another major source of carbon and energy for colonic bacteria. Though most dietary proteins are digested and absorbed in the small intestine, a load of unabsorbed proteins and peptides reaches the colon, where it serves as a substrate for fermentation by the resident bacteria. In the colon, protein breakdown takes advantage of the host’s endopeptidases and bacterial proteases (Cummings and Macfarlane, 1991). The proteolysis yields peptides and/or amino acids that can be subjected to Stickland reactions or other fermentation pathways by amino acid-fermenting bacteria (Smith and Macfarlane, 1998; Kim et al., 2004). Carbon dioxide, ammonia, and organic acids bearing the side chain of the amino acids are the primary products of amino acid fermentation (Barker, 1981; Riedel et al., 2017). The organic acids can further serve as electron acceptors or donors in the oxidation or reduction of other compounds (keto-acids, H_2_, unsaturated fatty acids) and can undergo decarboxylation and transamination reactions (Davila et al., 2013). Therefore, the range of fermentation products encompasses linear and branched organic acids (acetate, butyrate, propionate, valerate, isobutyrate, 2-methylbutyrate, isolavalerate, etc.), phenols and indole derivatives originating from aromatic amino acids, and a variety of nitrogen or sulfur-containing compounds (Smith and Macfarlane, 1996; Davila et al., 2013; Russell et al., 2013a). Unlike the saccharolytic catabolism, many of these products are undisputedly detrimental to health (in particular ammonia, phenols, indoles, amines, sulfides, and *N*-nitroso compounds), having a variety of harmful effects, including systemic toxicity, nephrotoxicity, and carcinogenesis (Blachier et al., 2010; Russell et al., 2013a, b; Barrios et al., 2015; Kobayashi, 2017).

A large amount of information on the human gut microbiota has been generated in recent years, favored by next-generation sequencing (NGS) technologies (Moore-Connors et al., 2016). NGS enabled and drove the detailed description of microbiota composition but the assessment of the functional role of bacteria was generally neglected. In recent years, many studies have been done on the effects of dietary protein on gut microbiota (Wu et al., 2011; David et al., 2014; Ma et al., 2017); nevertheless, most functional information on the bacteria involved in gut protein catabolism come from culture-based studies dependent on the isolation and physiological characterization of cultivable species (Cummings and Macfarlane, 1991; Smith and Macfarlane, 1998; Russell et al., 2013a). Clostridia and peptostreptococci are the most frequent isolates in media containing amino acids as energy and carbon sources, along with a wide range of bacteria belonging to *Fusobacterium*, *Bacteroides*, *Propionibacterium*, *Actinomyces*, Enterobacteria, and Gram-positive cocci (Davila et al., 2013). In the present study, fecal batch cultures with a medium containing only proteins and peptones as fermentable carbon sources were carried out in order to enrich the bacterial fraction taking advantage of protein fermentation. The progression of the microbiota was monitored by 16S rRNA gene profiling in order to identify the taxa involved in protein breakdown and fermentation.

## Materials and Methods

### Chemicals and Culture Medium

All the chemicals were purchased from Sigma-Aldrich (Steinheim, Germany) unless otherwise stated. The Protein Fermenter Medium (PFM) medium, developed by modifying Walker et al.’s (2005), contained 1.5 g/L sodium caseinate, 1.5 g/L beef extract, 1 g/L peptone (BD Difco, Sparks, NV, United States), 2 g/L KH_2_PO_4_, 4.5 g/L NaCl, 0.5 g/L MgSO_4_ ⋅ 7H_2_O, 0.045 g/L CaCl_2_ ⋅ 2H_2_O, 0.005 g/L FeSO_4_ ⋅ 7H_2_O, 0.01 g/L hemin, 0.05 g/L bile salts (Oxgall, BD Difco), 0.6 mg/L resazurin, and 0.2 mL/L antifoam (Xiameter 1520, Dow Corning, Midland, MI, United States). The following filter-sterilized solutions were added to the autoclaved medium: 2 mL/L minerals (500 mg/L EDTA, 200 mg/L FeSO_4_ ⋅ 7H_2_O, 10 mg/L ZnSO_4_ ⋅ 7H_2_O, 3 mg/L MnCl_2_ ⋅ 7H_2_O; 30 mg/L H_3_BO_3_, 20 mg/L CoCl_2_ ⋅ 6H_2_0, 1 mg/L CuCl_2_ ⋅ 2H_2_O, 2 mg/L NiCl_2_ ⋅ 6H_2_O, and 3 mg/L NaMoO_4_ ⋅ 2H_2_O), 1.4 mL/L vitamins (1 g/L menadione, 2 g/L biotin, 2 g/L calcium pantothenate, 10 g/L nicotinamide, 0.5 g/L cyanocobalamin, 0.5 g/L folic acid, 4 g/L thiamine, and 5 g/L PABA), and 40 mL/L reducing solution (12.5 g/L L-cysteine ⋅ HCl and 80 g/L NaHCO_3_).

### Fermentation Experiments

Fecal samples from six healthy volunteers (three men and three women aged 25–50 years) were collected fresh, having obtained written informed consent, according to the experimental protocol that was approved with ref. no. 125-15 by the local research ethics committee (Comitato Etico Provinciale, Azienda Policlinico di Modena, Italy). Subjects had not taken prebiotics and/or probiotics for the previous 2 weeks or antibiotics for at least 3 months prior to sample collection. The feces were homogenized 5% (w/v) with sterile PFM medium under an 85% N_2_, 10% CO_2_, 5% H_2_ atmosphere in an anaerobic cabinet (Anaerobic System, Forma Scientific, Marietta, OH). 60 or 3 mL of the suspension were utilized to seed PFM cultures with a concentrated or a diluted inoculation, 1 or 0.05% feces (w/v), respectively. Hereinafter, the fermentations that were inoculated at 1% are referred to as C (i.e., concentrated) processes, while the ones inoculated at 0.05% are referred to as D (i.e., diluted) processes.

Batch cultures with a volume of 400 mL were carried out in a multivessel bioreactor apparatus (Sixfors V3.01, Infors, Bottmingen, Switzerland), incubated at 37°C under a CO_2_ atmosphere (Bottari et al., 2017). The pH was maintained at 6.8 through automatic titration with 1 M HCl and 1 M NaOH. Samples for 16S rRNA gene analysis were collected at 0, 6, and 12 h of incubation.

A single preliminary fermentation run was carried out with subject V1, seeded with a C inoculation. Parallel fermentation runs were carried out for subject V2, both seeded with a C inoculation. For subjects V3, V4, V5, and V6, parallel fermentation runs were carried out with C and D inoculation conditions.

### DNA Extraction, 16S rRNA Gene Amplification, Library Construction, and Sequencing

Total DNA was extracted using a QIAamp DNA Stool Mini Kit (Quiagen, Hilden, Germany) following the manufacturer’s protocol. The DNA was quantified with a Qubit 3.0 fluorimeter (Thermo Fisher Scientific, Waltham, MA, United States) in order to normalize the template for 16S rDNA sequencing to 5 ng/μL.

Amplicons of approx. 460 bp were generated with PCR primers, targeting the V3 to V4 regions of the 16S rRNA gene and including Illumina adapter sequences (F-5′TCGTCGGCAGCGTCAGATGTGTATAAGAGACAGCCT ACGGGNGGCWGCAG, R-5′GTCTCGTGGGCTCGGAGATG TGTATAAGAGACAGGACTACHVGGGTATCTAATCC). The PCR reaction was performed in a 96-well Mastercycler-pro apparatus (Eppendorf, Hamburg, Germany) with the following program: 3 min at 95°C; 25 cycles of 30 s at 95°C, 30 s at 55°C, and 30 s at 72°C; 72°C for 7 min. An Illumina Nextera XT Index kit (Illumina Inc., San Diego, CA, United States) with dual 8-base indexes was used for multiplexing. The indexes were incorporated into the amplicons by means of PCR reactions with the following conditions: 3 min at 95°C; 25 cycles of 30 s at 95°C, 30 s at 55°C, and 30 s at 72°C; 72°C for 7 min. Both amplification reactions were followed by purification with AMPure XP beads (Beckman-Coulter, Brea, CA, United States).

The amplicons were quantified using the Qubit 3.0 fluorimeter, pooled at a concentration of 4 nM each, and sequenced by 2 × 300 bp paired-end sequencing on the MiSeq platform using a MiSeq v3 Reagent Kit (Illumina). Each run included a minimum of 5% PhiX DNA as an internal control.

### Sequence Analysis

Raw sequences were cleaned and filtered by size and quality using MOTHUR v.1.25.0 software (Schloss et al., 2009). Quality filtered sequences were processed with the QIIME2 pipeline (Version 2019.1) for closed-reference picking of amplicon sequence variants (ASVs), taxonomy assignment, collapsing into operational taxonomic units (OTUs), and diversity metrics computation (Bolyen et al., 2018). Closed-reference picking and taxonomic assignation were carried out utilizing SILVA SSU database release 132^1^ as a reference with the similarity threshold set at 0.97.

Alpha diversity indexes (number of observed taxa, Chao1, Shannon, and Pielou) were computed to evaluate within-sample richness and evenness at different taxonomic levels. Kruskal–Wallis test by ranks was carried out with the appropriate QIIME2 plugin for alpha diversity comparisons between groups (0, 6, and 12 h samples and C and D cultures), utilizing feature tables rarefied at the same number of reads.

Pairwise sample dissimilarity (beta diversity) was computed with Jaccard, Bray–Curtis, Canberra, Unweighted Normalized UniFrac, and Weighted Normalized UniFrac metrics resulting in distance/dissimilarity matrices, which enabled the hierarchical clustering of samples in UPGMA trees and Principal Coordinate Analysis (PCoA). Beta diversity comparisons within and between groups were carried through the analysis of similarities (ANOSIM) and permutational multivariate analysis of variance (PERMANOVA), using the appropriate QIIME2 plugin. The Linear discriminant analysis Effect Size (LEfSe)^2^ algorithm was applied to discover distinctive features at different time-points and to compare C and D cultures with the founding microbiota (Segata et al., 2011).

Spearman’s correlation was computed within the OTUs and between them and the concentration of ammonium, indole, and *p*-cresol. Before computing the correlation, sparsity reduction was applied on the feature table, keeping only the OTUs with at least 11 total reads (quantile 30%) and appearing in least in seven samples. The dataset was then rarefied using the smallest sample size. Sparsity reduction, rarefaction, and calculation of correlation were carried out with R software (version 3.6), with libraries “vegan” and “corrr.”

### Chemical Analysis

Acetate, lactate, propionate, butyrate, formate, succinate, and ethanol in the culture supernatants were quantified by HPLC with a refractive index detector (1200 System, Agilent Technologies, Waldbronn, Germany) and an Aminex HPX-87 H ion exclusion column. Isocratic elution was carried out at 60°C with 0.8 mL/min of 5 mM H_2_SO_4_ (Amaretti et al., 2013).

Indole and *p*-cresol were quantified by an HPLC (1100 System, Agilent Technologies) equipped with a diode array detector and a C18 Kinetex column (Phenomenex, Torrance, CA, United States). Elution was carried out at the flow of 0.8 mL/min, with a gradient of 0.1% (v/v) formic acid in water (eluent A) and 0.1% (v/v) formic acid in methanol (eluent B), eluent B increasing from 30 to 95% in 15 min and remaining at 95% till 20 min. Detection was carried out at 275 nm.

Ammonium was analyzed with a colorimetric assay. Briefly, 400 μL of properly diluted supernatant was mixed with 400 μL of Phenol nitroprusside solution (Sigma-Aldrich, P6994) and 400 μL of Alkaline Hypochlorite solution (Sigma-Aldrich, A1727) and incubated at room temperature for 30 min. Absorbance was read at 625 nm and compared with a calibration curve in the range from 0 to 150 μM.

Total carbohydrates were analyzed with anthrone colorimetric assay. 500 μL of culture was mixed with 1,000 μL of 2 g/L anthrone in H_2_SO_4_ 96%, incubated at 10 min at 100°C, then cooled in ice. Absorbance was measured at 620 nm and compared with a calibration curve in the range from 0 to 150 mg/L.

ANOVA followed by Tukey’s *post hoc* test was carried out to analyze the trend of metabolites over time in C and D fermentation, separately. Differences were considered statistically significant for *P* < 0.05. The *t*-test was utilized to compare the means of metabolite concentrations in C and D cultures at each time-point. Differences were considered statistically significant for *P* < 0.05. The statistical analysis was performed with IBM SPSS Statistics 21 software.

## Results

### Batch Cultures of Human Gut Microbiota in Protein-Based Medium

**FIGURE 1:**
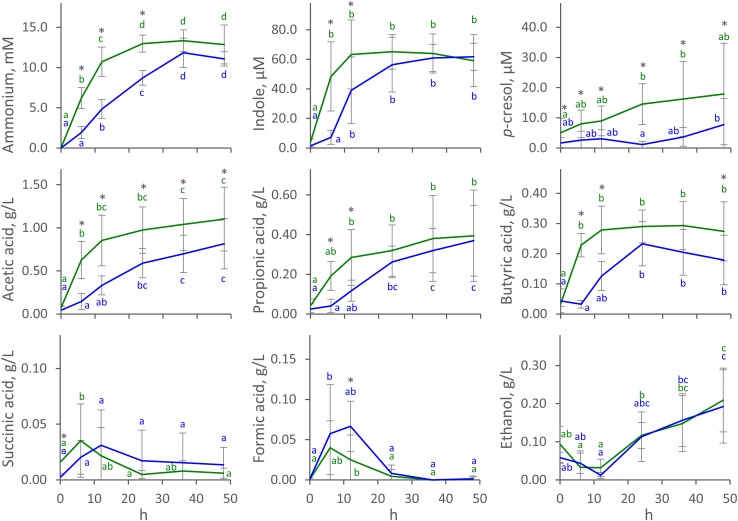
Time course of metabolites in C (green) and D (blue) cultures. Values are means ± SD (C, *n* = 8; D, *n* = 4). Within each series, means sharing the same letter did not significantly differ (ANOVA with Tukey *post hoc*; *P* < 0.05); C and D means significantly differing at the same time-point are indicated with ^∗^ (*t*-test; *P* < 0.05).

The time course of the concentration of metabolites in C and D cultures (mean) is reported in Figure 1, with single runs shown in Supplementary Figure S1. Ammonium was produced during all the fermentations. In C processes, it reached a stationary concentration of 13.0 ± 1.1 mM (mean ± SD) at 24 h, with most of the production (at least 75% of the final concentration) occurring in the first 12 h. Ammonium production was slower in D processes, which yielded a lower concentration at 24 h (8.7 ± 0.9 mM) compared to C processes (*P* < 0.05), and were protracted throughout the whole course of fermentation. Similar behavior was observed for indole, which reached a maximum of 63 ± 23 μM (mean ± SD) at 12 h in C fermentations but was produced more slowly in D runs, yielding 39 ± 22 μM at the same time-point (*P* < 0.05). Unlike ammonium and indole, *p*-cresol was produced mostly toward the end of the fermentation, being always more abundant in C than in D runs (*P* < 0.05). *p*-cresol was notably abundant in V2 processes, where it reached 40 μM at the end of the fermentation (Supplementary Figure S1).

Acetic, propionic, and butyric acids were the main organic acids that accumulated throughout the fermentations. In C fermentations, they had all already reached a stationary level at 6 or 12 h, while production was slower in D processes and reached stationary values at 12 or 24 h. In C fermentations, acetic, propionic, and butyric acids reached 0.85 ± 0.29, 0.28 ± 0.14, and 0.28 ± 0.08 g/L (mean ± SD) after 12 h, while significantly lower concentrations (*P* < 0.05) were observed in D fermentations (0.33 ± 0.11, 0.12 ± 0.05, 0.13 ± 0.05 g/L, respectively). Succinic and formic acids were also produced, although in low amounts. They transiently accumulated in the first 12 h, never increasing above 0.12 g/L, then decreased to negligible concentrations toward the end of the fermentation. Net ethanol production started at 12 h and continued to the end of fermentation, reaching up to 0.2 ± 0.1 g/L (mean ± SD). With regard to succinic acid, formic acid, and ethanol, no significant differences were observed between C and D processes (*P* > 0.05). Carbohydrates were always < 80 mg/L at 0 h and were depleted in the first 6 h.

### Diversity of Microbiota Cultures

The 16S rRNA gene profile of the cultures was determined at 0, 6, and 12 h, when most of the protein fermentation was observed. A total of 1,685,682 quality-trimmed 16S rRNA gene sequences were obtained from 29 samples, on average 58,127 reads per sample (Supplementary Datasheet S1). Sequences were dereplicated into 4,174 ASVs, hitting a reference sequence in the Silva database, and collapsed at the seventh level of taxonomic annotation (i.e., the species, if available) into 411 OTUs.

Richness (number of OTUs and Chao1 index) was higher at 0 h than at 6 and 12 h (*P* < 0.05), while the evenness (Shannon and Pielou indexes) did not change during the process (*P* > 0.05) (Supplementary Figure S2). Regardless of the incubation time, both the richness and the evenness were significantly higher in C than in D processes.

**FIGURE 2:**
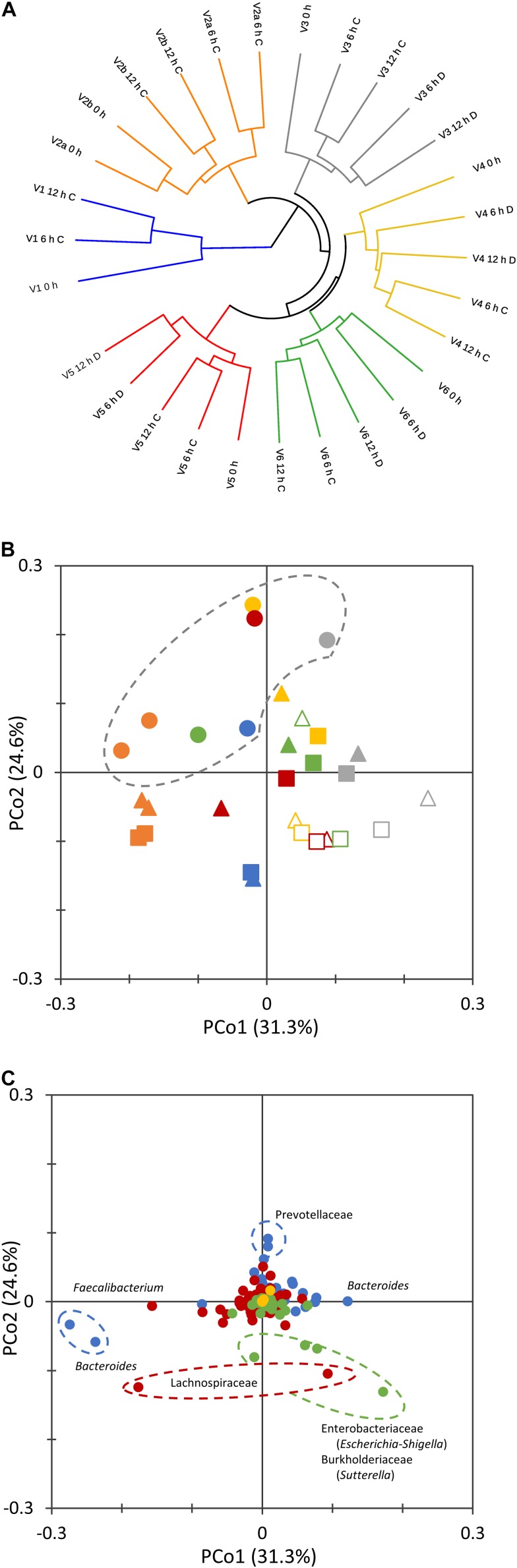
Beta diversity analysis of microbiota cultures at different times during cultivation. UPGMA dendrogram displaying Bray–Curtis distances, computed with the taxonomic features collapsed at the level of species **(A)**. 2D PCoA visualization of phylogenetic distances, computed as Normalized Weighted Unifrac **(B,C)**. Panel **(B)** reports the scores of samples from different subjects (V1, cyan; V2, orange; V3, gray; V4, yellow; V5, red; V6, green) at 0 (circle), 6 (triangle), and 12 h (square) of cultivation. Empty symbols correspond to D cultures. Panel **(C)** reports the contribution of single bacterial taxa. Bacteroidetes, blue; Firmicutes, red; Proteobacteria, green; Verrucomicrobia, yellow; other phyla, gray.

The non-phylogenetic beta diversity metrics evidenced subject-based clusters, with samples not grouping according to the time or the concentration of the inoculum (Figure 2A). With UniFrac metrics, which consider phylogenetic distances, groups based on the incubation time (0, 6, and 12 h) could be distinguished (Figure 2B). Time-based groups had different centroids (PERMANOVA, *P* = 0.002), and the similarity within each group was significantly higher than that between them (ANOSIM, *P* = 0.002). 0 h samples lay in the same region in the PCoA plot and similarly moved toward low PCo1 and high PCo2 values during the fermentation.

The twin processes seeded with the same amount of microbiota from subject V2 behaved similarly. Within the whole set of cultures, the distance between samples at 0 and 6 h was generally higher than that between 6 and 12 h. For the pairs of processes that were inoculated with different amounts of microbiota, D cultures evolved more distantly from the initial microbiota than did C cultures (*P* < 0.05).

The ASVs that mainly contributed to negative PCo2 belonged to the Enterobacteriaceae (*Escherichia-Shigella*), Burkholderiaceae (*Sutterella*), and Lachnospiraceae families, whereas Prevotellaceae were the main drivers of positive PCo2 (Figure 2C). Sequences ascribed to the genus *Bacteroides* highly contributed to both positive and negative PCo1, which was negatively driven also by *Faecalibacterium*.

### Microbiota Evolution and Analysis of Differential Abundances

**FIGURE 3:**
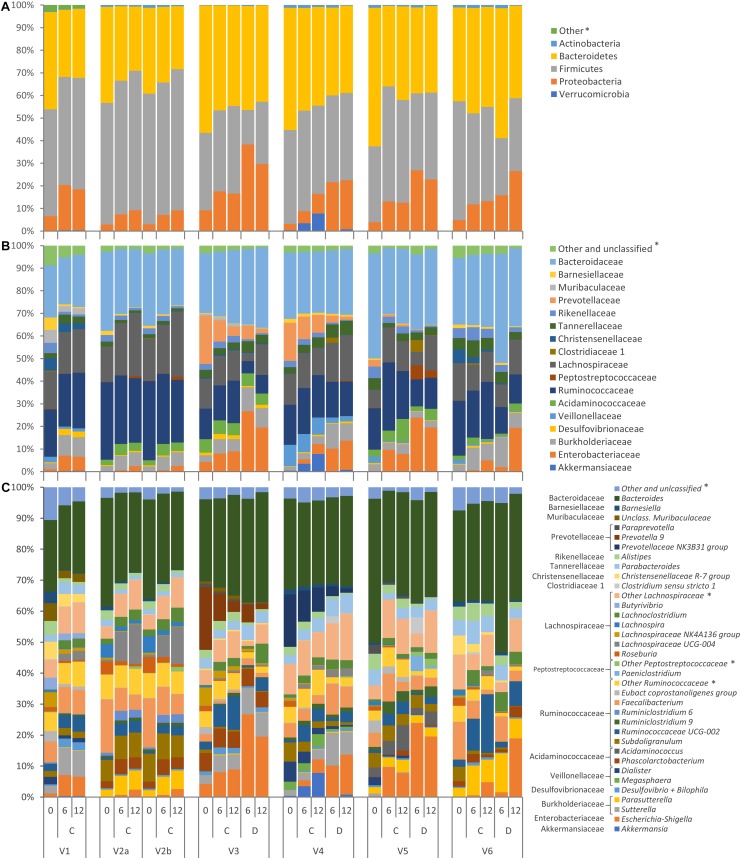
Stacked bar-plot representation of microbiota compositions during C and D batch cultivation, with taxonomic features collapsed at the level of phyla **(A)**, families **(B)**, and genera **(C)**. The phyla, families, and genera that remained unclassified or never occurred with abundance higher than 2.5% are grouped as others (^∗^).

**FIGURE 4:**
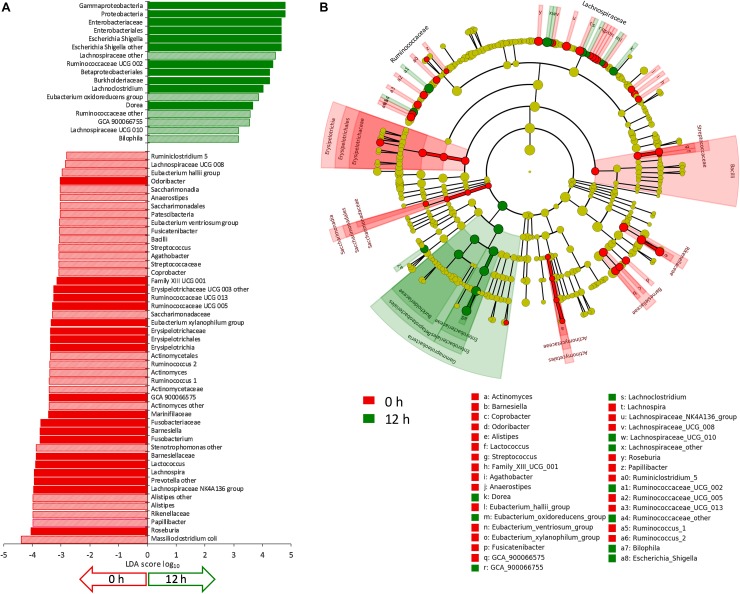
**(A)** LDA logarithmic scores of taxonomic biomarkers found by LEfSe exhibiting significant differential abundance (*P* < 0.05, logarithmic LDA score ≥ 2.0) between 0 and 12 h, regardless of inoculation load (C and D processes). Filled bars represent the taxa with significant differential abundance already after 6 h. **(B)** Cladogram visualization of the taxonomic biomarkers characterizing 0 and 12 h.

The evolution of microbiota is reported in Figure 3. A total of 64 taxa exhibited significant difference between 0 and 12 h (Figure 4), 17 becoming enriched and 47 decreasing. In most cases, significant enrichment was already observed after 6 h. Proteobacteria and taxa ascribed to Ruminococcaceae and Lachnospiraceae were the main biomarkers of the grown cultures, regardless of inoculation load. Comparison between C and D cultures revealed that Firmicutes characterized the former and Proteobacteria the latter (Supplementary Figure S3). The whole phylum Proteobacteria, the class Gammaproteobacteria, and the orders Enterobacteriales (with Enterobacteriaceae) and Betaproteobacteriales (with Burkholderiaceae) significantly increased regardless of the inoculum load but became significantly more abundant in D cultures than in C cultures. At the level of genus and species, the Enterobacteriaceae *Escherichia-Shigella* and the Desulfovibrionaceae *Bilophila* always characterized the grown cultures, while Xanthomonadales, such as *Dyella* and *Stenotrophomonas maltophila*, presented a significant increment only in D cultures. Within Firmicutes, some Lachnospiraceae and Ruminococcaceae (such as *Dorea*, *Eubacterium oxidoreducens, Lachnoclostridium*, and other uncultured/unclassified members) were among the most significantly enriched taxa. Their increase was significant regardless of inoculum dilution, but they became more abundant in C cultures. In particular, the increase of the Lachnospiraceae *Sellimonas* was observed only in C cultures, together with the Ruminococcaceae *Anaerotruncus* and *Oscillibacter*. Within Actinobacteria, Coriobacteriaceae and particularly *Collinsella* presented significant increments, but only in D cultures.

Other taxa showed a drastic increase and reached remarkably high levels but were not identified as biomarkers by LEfSe because they occurred only in a few founding microbiota or because they thrived only in some of the cultures where they were initially present. Numerous taxa of Proteobacteria increased during the incubation, in some cases starting from values initially lying below the limit of detection, but were detected in a minority of samples. For example, *Sutterella* was abundant in cultures V1, V3, and V4, where it increased, particularly in D processes, while *Parasutterella* was abundant and increased in V2, V5, and V6 cultures. Peptostreptococcaceae were initially negligible in all the cultures and did not present any significant change. Interestingly, they became abundant in the D cultures of V5, where they increased by two magnitudes to 6.3%, mainly because of the contribution of *Paeniclostridium* and *Romboutsia*. Among Clostridiaceae, only *Clostridium sensu stricto* 1, generally absent or negligible at 0 h, became enriched in D processes inoculated with the feces of subject V5 by more than two magnitudes, reaching 5.3%. *Acidaminococcus* (fam. Acidaminococcaceae) was absent in most of the cultures and abundant only in V5, where it grew considerably. The Verrucomicrobia *Akkermansia muciniphila* was initially absent or scarce in all of the cultures, and it reached a remarkably high abundance only in V4 cultures, in particular C, where it became one of the dominant taxa (7.8%).

Other taxa did not respond positively to proteinaceous substrates. Bacteroidaceae, almost coinciding with the genus *Bacteroides*, did not significantly change, while other Bacteroidetes, such as Rikenellaceae (including the genus *Alistipes*), Barnesiellaceae (such as *Barnesiella* and *Coprobacter*), and Marinifilaceae (including the genus *Odoribacter*) significantly decreased. Prevotellaceae were initially remarkably abundant only in V3 and V4 and drastically decreased. Among Firmicutes, many members of Rumicococcaceae and Lachnospiraceae (such a *Papillibacter*, *Ruminiclostridium*, *Ruminococcus*, *Subdoligranulum*, *Agathobacter*, *Anaerostipes*, *Fusicatenibacter*, *Lachnospira*, *Roseburia*, *Eubacterium xylanophilum*, *Eubacterium ventriosum*, *Eubacterium hallii*, and other unclassified members) significantly decreased during cultivation or presented a tendency to decrease (*Butyrivibrio*). Also Bacilli (such as Streptococcaceae, *Streptococcus*, and *Lactococcus*) and Erysipelotrichaceae significantly diminished, while Acidaminococcaceae (*Dialister*), Christensennellaceae, and Vellionellaceae presented a negative tendency, in particular in the cultures where their relative abundance was initially remarkably high (*Dialister* in V4; Christensennellaceae in V1 and V6; Vellionellaceae in V1, V4, and V5). The abundance of the saccharolytic bifidobacteriaceae significantly decreased.

### Correlation Analysis

**FIGURE 5:**
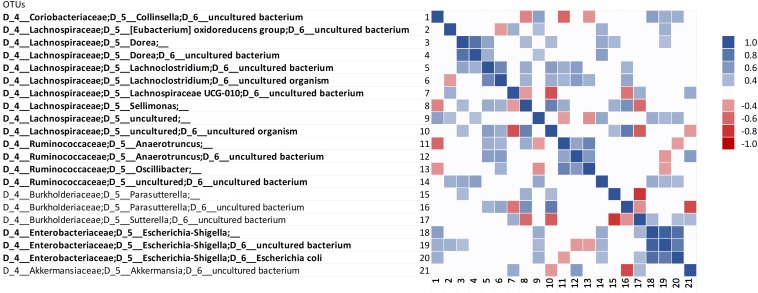
Spearman’s rank correlation among the OTUs that positively responded to proteinaceous substrates. Rho values are reported in the heatmap if statistically significant (*P* < 0.05), ranging from the deepest red (–1) to the deepest blue (+1). The OTUs that were evidenced by LEfSe as biomarkers with significant increase are in bold.

Correlation analysis was carried out within the 158 most abundant OTUs and between them and the concentration of ammonium, indole, and *p*-cresol. Correlation analysis among the OTUs did not identify evident clusters (Supplementary Datasheet S2). However, the Bacteroidetes were generally in positive correlation with each other and in negative correlation with a variety of Firmicutes, including taxa that presented different responses to proteins (e.g., *Lachnoclostridium* and *Subdoligranulum*). Restricting the analysis to the OTUs that positively responded to proteinaceous substrates confirmed the predominance of different biomarkers in C and D cultures (Figure 5). In fact, positive correlations were observed within the biomarkers of grown D (*Collinsella*, *Esherichia-Shigella*, and *Escherichia coli*) or C cultures (*Anaeortruncus*, *Oscillibacter*, and *Sellimonas*), whereas negative correlations occurred between C and D biomarkers. The putatively proteolytic OTUs of Lachnospiraceae and Ruminococcaceae were generally in positive correlation with each other, with only a few cases of negative correlations. Some negative correlations were also observed between putative proteolytic Firmicutes and Enterobacteria, mainly *Sutterella*, *Parasutterella*, and *Escherichia-Shigella*. *Sutterella* and *Parasutterella* were mutually exclusive in the samples inoculated with different microbiota.

**FIGURE 6:**
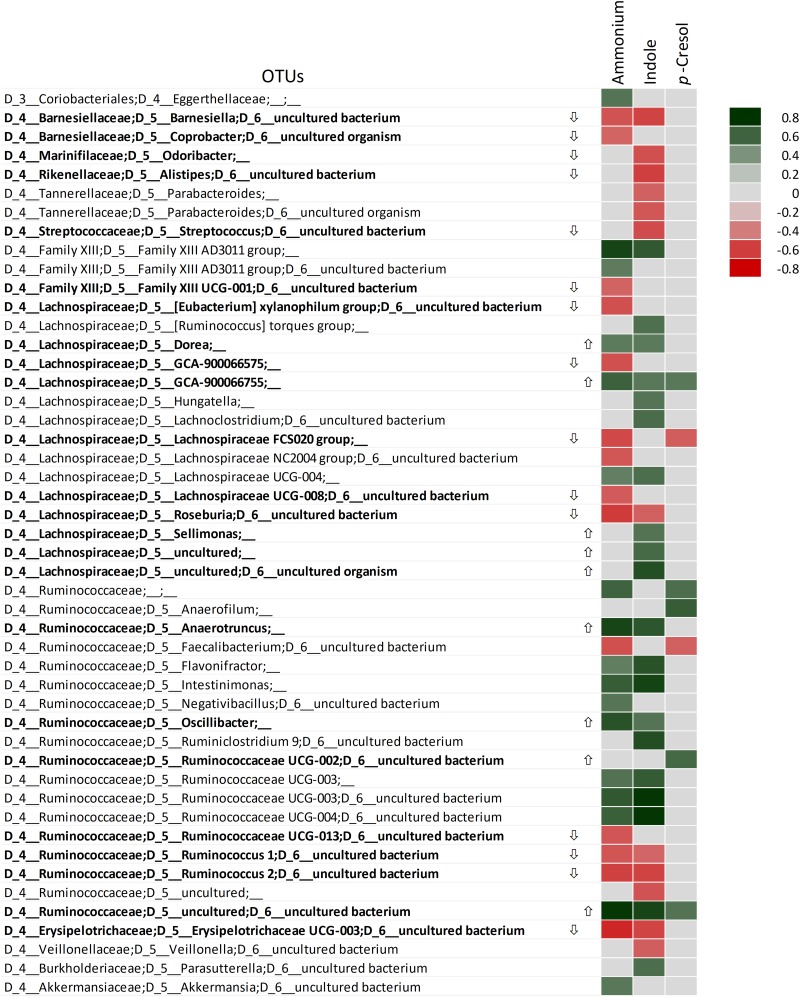
Spearman’s rank correlation among the OTUs and ammonium, indole, and *p*-cresol. Only OTUs significantly correlating with at least one metabolite are displayed. Rho values are reported in the heatmap if statistically significant (*P* < 0.05), ranging from the deepest red (–1) to the deepest green (+1). The OTUs that were evidenced by LEfSe as biomarkers with significant increase (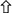) or decrease (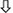) are in bold.

Positive correlations with ammonium, indole, and/or *p*-cresol, were observed for OTUs attributed to Ruminococcaceae (13 OTUs), Lachnospiraceae (11 OTUs), Family XIII Clostridiales (2 OTUs), and Eggerthellaceae, *Akkermasia*, and *Parasutterella* (1 OTU each) (Figure 6). Some of them were previously identified as growth biomarkers by LEfSe (e.g., *Anaerotruncus*, *Oscillibacter*, *Dorea*, *Sellimonas*) or presented a positive response to proteins in some cultures (e.g., *Akkermasia*, and *Parasutterella*). Positive correlation with metabolites was also detected for some taxa that were not previously identified, such as some Lachnispiraceae (*Hungatella* and *Ruminococcus torques*, etc.), Ruminococcaceae (*Anaerophilum*, *Flavonifractor*, *Intestinimonas*, *Negativibacillus*, *Ruminiclostridium*, etc.), Family XIII Clostridiales, and Eggerthellaceae. On the other hand, the OTUs of Proteobacteria that became enriched during cultivation did not exhibit any significant correlation with ammonia, indole, or *p*-cresol, with the exception of *Parasutterella*, which positively correlated with indole.

None of the OTUs that significantly decreased presented any positive correlation with ammonium, indole, or *p*-cresol. On the other hand, many taxa presenting a significant negative response to proteinaceous substrates negatively correlated with ammonium and indole. This is the case with Barnesiellaceae (such as *Barnesiella* and *Coprobacter*), Marinifilaceae (including the genus *Odoribacter*), and several OTUs of Lachnospiraceae and Ruminococcaceae, such as *Roseburia*, *Ruminococcus*.

## Discussion

The occurrence of protein catabolism by the gut microbiota was established by early culture-based works that identified *Bacteroides* and *Propionibacterium* as dominant proteolytic species and recognized proteolysis as common amongst clostridia, streptococci, staphylococci, and *Bacillus* species (Macfarlane et al., 1986; Smith and Macfarlane, 1998; Davila et al., 2013). However, proteolysis is a wide concept that can refer to the hydrolysis of proteins to provide microorganisms with a nitrogen source or to proper fermentation of amino acids to obtain energy and C-precursors. The bacterial utilization of proteins involves a complex metabolic network starting with the hydrolysis to peptides and amino acids, which can be assimilated in anabolic processes and/or fermented to gain energy and C-sources.

The gut microbiota enrichment cultures herein described aimed to identify, through a 16S rRNA gene survey, the bacterial taxa that take advantage of proteins, peptones, and amino acids as the sole available carbon sources. The approach relied on registering differences in relative bacterial amounts during pH-controlled batch processes, with the awareness that a major increase in some groups may conceal a lesser increase in others. Based on the accumulation of organic acids, ammonia, and indole, the cultures successfully selected bacteria performing protein breakdown and fermentation. In the complex relationship of nutritional cross-feeding interactions that characterizes the gut microbiota, true protein and amino acid fermenters became enriched, especially in the first hours of the process, likely accompanied or followed by bacteria utilizing the fermentation products.

Despite differences in the founding communities, the cultures described similar trajectories in the PCo space of Weighted Normalized Unifrac, suggesting that they evolved in the same direction and shared enrichment in phylogenetically similar bacterial groups. Analysis of differential abundances among time-points leads to the identification of taxa participating in protein breakdown and fermentation, particularly within the Enterobacteriaceae, Burkholderiaceae, Ruminococcaceae, and Lachnospiraceae. Other groups, some characterized by saccharolytic metabolism, clearly decreased in relative abundance, for example many Firmicutes (e.g., *Roseburia*, *Ruminococcus*, *Lachnospira, Dialister*, and Erysipelotrichaceae) and Bacteroidetes (e.g., Barnesiellaceae, Prevotellaceae, and Rickenelliaceae).

Despite D cultures being carried out to detect any growth even in the most concentrated taxa, no significant changes in the relative abundance of Bacteroidaceae and related taxa were observed. *Bacteroides* are major primary degraders of oligo- and polysaccharides (Wexler, 2007). However, members of this genus also produce extracellular proteases and exopeptidases with a variety of arylamidase activities (Riepe et al., 1980; Macfarlane and Macfarlane, 1997; Song et al., 2004), ferment amino acids (Smith and Macfarlane, 1998), and positively correlate with the consumption of protein in human and animal studies (Wu et al., 2011; Rist et al., 2014). In the present study, a wide number of ASVs attributed to *Bacteroides* presented opposite contributions along the principal coordinates. It is plausible that different populations of *Bacteroides*, which could not be discriminated into species by the partial 16S rRNA gene sequencing, behaved differently with respect to protein breakdown, exerting contrary effects and balancing the counts of the genus. As a whole, by virtue of the great abundance in the gut, *Bacteroides* likely maintain a pivotal role as primary protein hydrolyzers, making amino acids and peptides available for both biosynthesis reactions and catabolism by a larger number of bacteria, ranging from facultative saccharolytic bacteria to obligate amino acid fermenters.

Because of their easy isolation and cultivation, Enterobacteriaceae such as *E. coli, Enterobacter*, *Klebsiella*, and *Shigella* spp. were among the first gut bacteria to be identified as protein degraders, in agreement with the results of this study, which identified all the taxonomic levels of Proteobacteria as biomarkers of growth on proteins. Enterobacteriaceae, and in particular *E. coli*, are pathobionts bearing a number of virulence genes that may turn from commensal organisms into pathogens responsible for severe diseases when specific genetic or environmental conditions are altered in the host (Raimondi et al., 2019). High levels of *E. coli* have been associated with inflammatory bowel disease and cancer (Veziant et al., 2016), and thus the proliferation of *E. coli* and Enterobacteriaceae may promote inflammation, determine colitogenic effects, and favor the growth of subdominant populations playing a role in carcinogenesis.

Correlation analysis pointed out members of Ruminococcaceae and Lachnospiraceae as main producers of the harmful metabolites ammonium, indole, and *p*-cresol, with several of these OTUs ascribed to uncultured bacteria or to genera not investigated in terms of metabolism, physiology, and relationship with the host. Interestingly, Enterobacteriaceae and *E. coli*, proteolytic bacteria regarded as major indole producers (Lee and Lee, 2010; Dai et al., 2011), did not present significant correlations with this metabolite or with ammonium and *p*-cresol. As a matter of fact, all of these metabolites reached higher concentrations in C cultures, whereas several taxa of Proteobacteria, including *E. coli*, bloomed mainly in D cultures, where protein metabolites were less concentrated.

Burkholderiaceae and Desulfovibrionaceae also responded positively to proteinaceous substrates, with an increase of asaccharolytic anaerobes/microaerophiles such as *Sutterella*, *Parasutterella*, and *Bilophila* (Wexler et al., 1996; Ju et al., 2019). These genera are core components of the human gut microbiota, occurring ubiquitously, but are still understudied in terms of physiology and role in the intestinal ecosystem. The present study demonstrated that they take advantage of the breakdown of proteinaceous substrates, providing one of the first insights into their role in the colonic ecology. However, it remains to be investigated whether these asaccharolytic bacteria grew as true amino acid fermenters or utilized the organic acids released by other bacteria (da Silva et al., 2008).

Several other taxa, which are still poorly characterized at the physiological level, emerged as participating in protein catabolism, for some species representing one of the first sources of functional information. Many bacterial biomarkers herein identified are members of Lachnospiraceae, Ruminococcaceae, and Peptostreptococcaceae (such as *Anaerotruncus*, *Dorea*, *Oscillibacter*, *Eubacterium oxidoreducens*, *Lachnoclostridium*, *Paeniclostridium*, *Rombutsia*, and other unclassified members), for which no information is available or very little is known on their possible role in protein catabolism and amino acid fermentation, apart from some correlation with a protein-rich diet (Taras et al., 2002; David et al., 2014; La Reau and Suen, 2018).

On the other hand, the opportunistic pathogen *Acidaminococcus* was already known as a true amino acid fermenter that is capable of utilizing amino acids as its sole carbon/energy source (Kim et al., 2004; D’Auria et al., 2011). *Acidaminococcus*, rarely detected in the founding microbiota, was particularly abundant and grew only in the cultures inoculated with a single founding microbiota.

Similar behavior was observed for the mucus degrader *Akkermansia*, which was negligible in most cultures but increased abundantly in the cultures of a sole subject. *A. mucinifila* is claimed as a next-generation probiotic because of the protective effect exerted against metabolic disorders and inflammatory diseases (Cani and de Vos, 2017). It is plausible that proteases are involved in the degradation of mucin and may have supported the growth in the protein-based microbiota cultures.

## Conclusion

This study shed light on the bacterial taxa thriving in cultures of human microbiota and utilizing proteins and peptides as their primary substrate for growth. It provided the first information on some enigmatic ubiquitous commensal bacteria. Protein catabolism is claimed to be detrimental for human health on the basis of metabolites like indoles and phenols. In terms of bacterial population, Proteobacteria, including Enterobacteriaceae and *E. coli*, are among the microbial group that most benefit from protein as C-sources, increasing the risk of the flourishing of opportunistic pathogens or bacteria associated to gut inflammation. However, Lachnospiraceae and Ruminococcaceae, that encompass a number of health-promoting saccharolytic bacteria such as the butyrate producers, presented several OTUs of proteolytic bacteria and showed the strongest correlation with the production of ammonium, indole, and *p*-cresol.

## Data Availability Statement

16S rRNA gene sequences have been submitted to NCBI repository with the BioProject ID: PRJNA540787.

## Ethics Statement

This study was carried out in accordance with the recommendations of the protocol approved with ref. no. 125-15 by Comitato Etico Provinciale, Azienda Policlinico di Modena, with written informed consent from all subjects. All subjects gave written informed consent in accordance with the Declaration of Helsinki. The protocol was approved by Comitato Etico Provinciale, Azienda Policlinico di Modena, Italy.

## Author Contributions

AA, AM, and MR conceived and designed the experiments. CG, MS, SR, and LR carried out the fermentations and chemical analysis. AA, VP-B, RG-L, and AM performed the metagenomic study and the bioinformatics. AA and MR wrote the manuscript with contributions from all other authors.

## Conflict of Interest

The authors declare that the research was conducted in the absence of any commercial or financial relationships that could be construed as a potential conflict of interest.

## Footnotes

1 https://www.arb-silva.de/download/arb-files/

2 http://huttenhower.sph.harvard.edu/galaxy
