# Supplementary material for: Profiling of Protein Degraders in Cultures of Human Gut Microbiota
Source: Front Microbiol. 2019 Nov 15;10:2614. doi: 10.3389/fmicb.2019.02614 (PMC6874058; doi:10.3389/fmicb.2019.02614)
Supplement: Supplementary file 3 [file Data_Sheet_3.docx]

Supplementary Material

Profiling of protein degraders in cultures of human gut microbiota

Alberto Amaretti, Caterina Gozzoli, Marta Simone, Stefano Raimondi, Lucia Righini,

Vicente Pérez-Brocal, Rodrigo García-López, Andrés Moya, Maddalena Rossi*

* Correspondence: Maddalena Rossi, [maddalena.rossi@unimore.it](mailto:maddalena.rossi@unimore.it)

**Supplementary figure S1**. Time-course of the batch cultures of human gut microbiota from subjects V1, V2, V3, V4, V5, and V6. Left: ethanol (green) and formic (orange), acetic (grey), propionic (yellow), butyric (blue), and succinic (red) acids. Right: ammonium (blue), indole (orange), and *p*-cresol (grey). Solid and dashed lines indicate C and D fermentations, respectively.


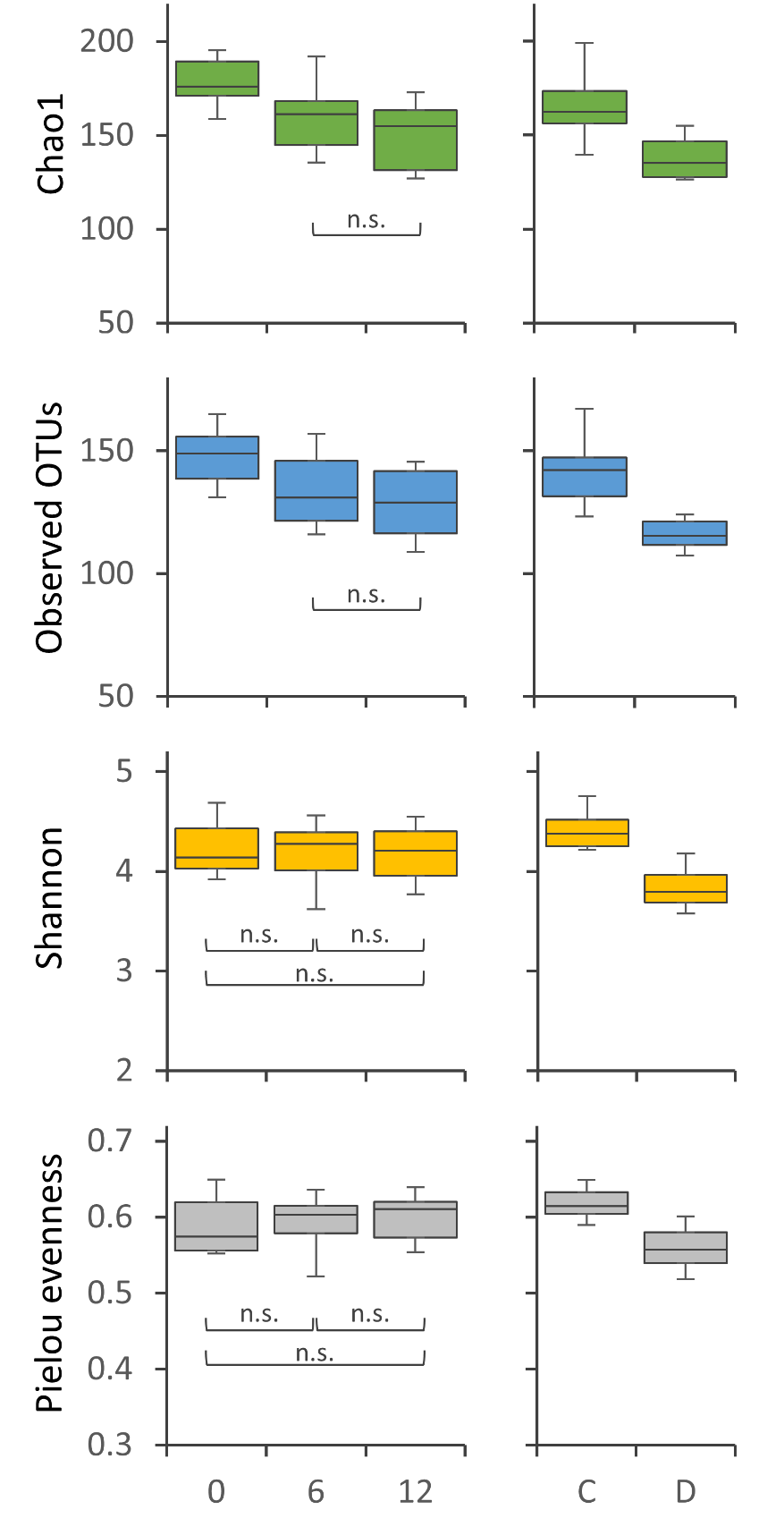


**Supplementary figure S2**. Alpha diversity indices (Caho1, Observed OTUs, Shannon, and Pielou) of microbiota cultures at different times of cultivation (left) and in C and D cultures. Indices were computed on feature tables rarefied at the same number of reads. Within each plot, groups significantly differ (Kruskal Wallis test, P < 0.05) unless otherwise stated (n.s.).

**Supplementary figure S3.** Panel A: LDA logarithmic scores of taxonomic biomarkers found by LEfSe, exhibiting significant differential abundance (P < 0.05, logarithmic LDA score ≥ 2.0) among 0 h, C (6 and 12 h), and D (6 and 12 h) samples. Filled bars represent the taxa with significant differential abundance already evidenced by the comparison of different time points regardless of C/D inoculation (Fig. 4). Panel B: Cladogram visualization of the taxonomic biomarkers.
